# A systematic review and meta-analysis of outcomes between dusting and fragmentation in retrograde intrarenal surgery

**DOI:** 10.1186/s12894-023-01283-w

**Published:** 2023-07-07

**Authors:** Zhi Wen, Li Wang, Yang Liu, Jing Huang, Cai-Xia Chen, Chong-Jian Wang, Lin-Lin Chen, Xue-song Yang

**Affiliations:** 1 Department of Urology, Affiliated Hospital of North Sichuan Medical College, Nanchong, Sichuan China; 2 Department of Hemodialysis, Sixth People’s Hospital of Nanchong, Nanchong, Sichuan Province China

**Keywords:** Ureteroscopy^1^, Nephrectomy^2^, Calculi^3^, Lithotripsy ^4^, Surgery^5^

## Abstract

**Objectives:**

Comparing stone-free rates and associated outcome measures between two surgical modalities of lithotripsy fragmentation and removal or spontaneous passage of dust during retrograde intrarenal surgery (RIRS).

**Methods:**

In March 2023, we conducted a literature search in several widely used databases worldwide, including PubMed, Embase, and Google Scholar. We only considered English articles and excluded pediatric patients. Reviews and protocols without any published data were excluded. We also excluded articles with conference abstracts and irrelevant content. We used the Cochran-Mantel–Haenszel method and random-effects models to assess inverse variances and 95% confidence intervals (CIs) for mean differences in categorical variables. The results were reported as odds ratios (ORs) and 95% CIs. Statistical significance was set at *p* < 0.05.

**Results:**

Our final meta-analysis included nine articles, comprising two randomized controlled trials (RCTs) and seven cohort studies. The total number of patients included in these studies was 1326, and all studies used holmium laser lithotripsy. The pooled analysis of the dust and fragmentation groups showed that the fragmentation group had a higher stone-free rate (OR 0.6; 95% CI 0.41 – 0.89; *p* = 0.01); the dust group had a shorter operative time (WMD – 11.6 min; 95% CI – 19.56 – –3.63; *p* = 0.004); and the dust group had a higher retreatment rate (OR 2.03; 95% CI 1.31 – 3.13; *p* = 0.001). There was no statistically significant difference between the two groups in terms of length of hospital stay, overall complications, or postoperative fever.

**Conclusions:**

Our results showed that both procedures could be safely and effectively used for upper ureteral and renal calculi lithotripsy, the dust group had potential advantages over the fragmentation group in terms of the operation time, and the fragmentation group had certain advantages in terms of stone-free rate and retreatment rate.

## Introduction

Kidney and ureter calculi are common urological diseases. Patients with kidney calculi usually have no symptoms, unless the calculi fall from the kidney into the ureter and cause urinary obstruction in the ureter. Common symptoms include lumbar and retroperitoneal pain, hematuria, and other symptoms. The incidence of this disease has been steadily increasing, resulting in a growing patient population [1]. Urinary calculi can arise from various factors, such as living environment, race, gender, and dietary habits, and can result from urinary tract obstruction, metabolic abnormalities, and infection. Crystal formation due to excessive urine concentration can eventually lead to stone deposition and aggregation, causing obstructive symptoms that significantly impact patients’ quality of life [2].

Urolithiasis treatment methods vary depending on the size of the calculi. In general, calculi smaller than 0.6 cm do not require surgical intervention, and only medical treatment, adequate water intake, and physical exercise are needed to help the calculi pass naturally. Treatment options for upper ureteral and kidney stones smaller than 2 cm include extracorporeal shock wave lithotripsy(ESWL), Ureteroscopic lithotripsy, open surgery, laparoscopic stone extraction, and percutaneous antegrade ureteroscopy [3]. The use of ureteroscopic techniques in the clinical treatment of upper urinary tract stones was first reported by Goodman in the 1970s [4]. Since then, flexible ureteroscopic lithotripsy has become an effective treatment for upper ureteral and renal calculi, thanks to the development of flexible ureteroscopes with a smaller diameter and significant changes in laser technology. The holmium laser, with a high efficiency and small diameter (200 µm) flexible laser fiber, can smoothly reach the upper ureter and cross the ureter to reach any part of the calyceal system [5]. The European Association of Urology (EAU) guidelines recommend retrograde intrarenal surgery (RIRS) as the first-line treatment for renal stones less than 2 cm and upper ureteral stones, However, PCNL is still the preferred approach for larger kidney calculi (> 2 cm) [6]. There are two primary surgical approaches to RIRS for ureteral and renal calculi: active removal of fragments using a basket or fragmentation of fragments into dust using a holmium laser to allow spontaneous passage. High-frequency and low-energy holmium laser cause stones to break into punctate fragments, a procedure called dusting. Dust formed under these conditions is typically left in place and excreted spontaneously by the urethra without the need for additional measures. In contrast, high-energy and low-frequency holmium laser lithotripsy, which breaks stones into larger fragments, a process called fragmentation, uses a basket or grasper to remove larger fragments during surgery to help patients remove stones. The potential advantages of dusting include shorter operative times and lower operative costs. While fragmentation offers potential benefits such as improved stone clearance and reduced risk of residual stone fragments leading to subsequent treatment events, there is currently no standardized optimal surgical approach for fragment treatment after laser lithotripsy. Even the Endourology Excellence Panel (EDGE) consortium did not reach a clear consensus on the best approach [7].

We aimed to systematically review the safety and stone-free rate after retrograde intrarenal surgery (RIRS) by comparing dusting with fragmentation techniques.

### Literature search

This study adhered to the standards specified in PRISMA [8] (Preferred Reporting Items for Systematic Reviews and Meta-Analysis) and was prospectively registered in the PROSPERO database (CRD42023411427). Articles included in the systematic review were investigated independently by two reviewers (WL and LY). The data obtained from the literature were before March 1, 2023. Extensive literature searches were conducted using MEDLINE, EMBASE, and Google Scholar databases. The search was limited to English language papers. Medical Subject Headings (MeSH) terms and keywords, such as "Ureteroscopy", "Calculi OR Kidney Calculi", "Lithotripsy, Laser OR Laser", "dust", "fragment", and "basket" In addition, we manually searched and reviewed relevant references to avoid any omission.

### Inclusion and exclusion criteria

The PICOS (Patient, Intervention, Comparison, Outcome, Study Type) model is used to construct and answer clinical questions. P (patients): Adult patients with renal and upper ureteral calculi who underwent RIRS with laser lithotripsy. I (intervention): Holmium laser fragmentation of ureteral calculi into dust to allow spontaneous passage.

C (comparison): calculi fragmentation into pieces and active removal using a basket or other tool. O (outcome): Operation time, postoperative hospital stay, overall complications, major complications, number of fevers, the incidence of postoperative hematuria, and stone clearance rate. and S (study), Cohort studies, case–control studies, and randomized controlled trials (RCTs). The exclusion criteria were as follows: (1) non-English articles; (2) non-comparative studies and (3) conference abstracts, case reports, letters, and any other unpublished articles.

### Study screening and selection

Two independent authors (WL and LY) manually screened all retrieved records. When consensus could not be reached between the two authors, it was resolved by consultation with a third author (WCJ). Papers were selected for screening by reading the full text if found to be relevant to the objectives of this study.

### Data items

Data were extracted independently by two reviewers, encompassing general information such as first author, publication year, and country, as well as population characteristics such as age, gender, and follow-up time, stone characteristics such as size, location, laser type, and laser setting parameters, and perioperative outcomes such as operation time, length of hospital stay, stone-free rate, postoperative fever, postoperative urinary tract infection, overall complications, and reoperation rate.

### Statistical analysis

In this study, statistical analysis was performed using Review Manager V5.4.1 software (Cochrane Collaboration, Oxford, UK). Results were presented with 95% confidence intervals (ci) and odds ratios (OR) for dichotomous variables and weighted mean difference (WMD) for continuous variables. Data from some studies reporting only medians, quartiles, or extreme value ranges were converted to means and standard deviations (SDs) using data conversion tables provided by McGrath [9]. Meta-analysis was performed using the Mantel–Haenszel method for dichotomous variables and the inverse variance method for continuous variables. Because of the predictable significance of heterogeneity across trials, random-effect models were used for all analyses. Study heterogeneity was calculated using the i [2] statistic, which defined 0 – 40% as mild heterogeneity; 40% – 60% as moderate heterogeneity; 50 – 90% as large heterogeneity; and 75 – 100% as great heterogeneity [10]. A value of *p* < 0.05 was considered statistically significant.

### Bias risk assessment

The Cochrane Risk of Bias assessment tool was used to evaluate the risk of bias in the included RCT studies. The tool assessed 5 domains for randomized controlled trials: randomization process (selection bias), over-intervention (implementation bias), missing data (loss to follow-up bias), outcome measurement (measurement bias), and outcome selection (reporting bias), which were categorized as low risk, unknown risk, and high risk [11]. For non-RCT studies, the Newcastle–Ottawa Scale (NOS) was used to evaluate the risk of bias. The quality of the literature was evaluated using a semiquantitative star system, which consisted of 9 stars.

### Sensitivity analysis

We used the leave-one-out method to exclude studies from the pooled effect one at a time to assess the robustness of the estimates. Furthermore, we evaluated the robustness based on the study cohort size (excluding studies with < 100 patients), which may contribute to heterogeneity. However, we cannot perform sensitivity analyses comparing three or fewer studies.

### Publication bias

When 10 or fewer studies were included, the power of the test was insufficient. Therefore, we did not perform further publication bias analysis [12, 13]

## Result

### Baseline characteristics

**Fig. 1:**
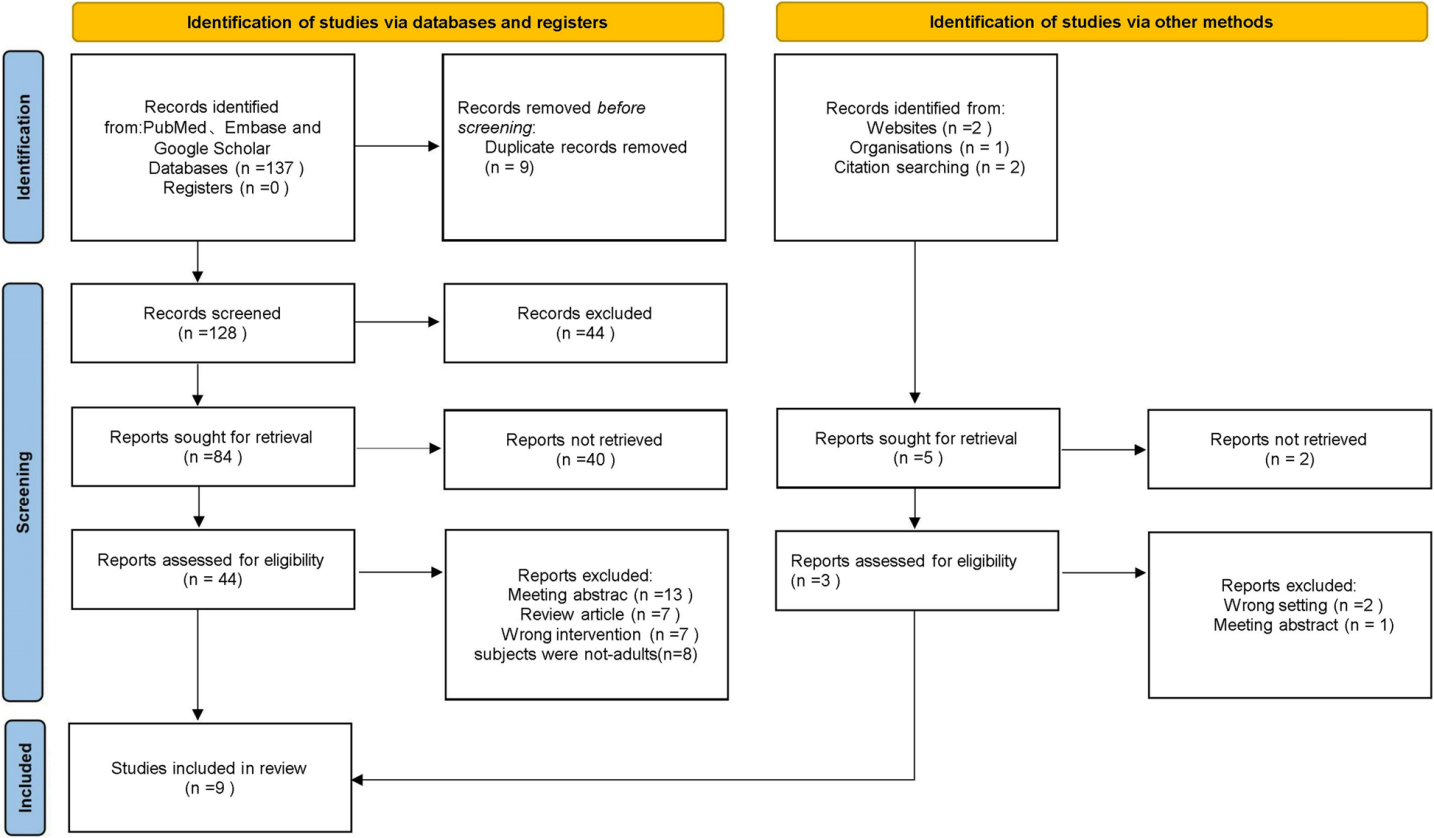
PRISMA flowchart

The initial literature search retrieved a total of 142 articles. After removing 5 duplicates, 137 studies remained for screening. Ninety-five of these papers were excluded from title and abstract screening because they were not relevant to the objectives of this study. The full texts of the remaining 47 studies were screened and 38 papers lacking data specificity, subjects were not adults, wrong interventions, etc., were further excluded. Finally, nine studies were accepted and included in the meta-analysis. These studies were conducted in different countries, including the United States of America (USA), Turkey, Israel, Kuwait, China, and Romania. Nine studies compared dusting and fragmentation methods in RIRS for the treatment of kidney stones and upper ureteral stones. There were 7 cohort studie [14–20] and 2 randomized clinical trials [21, 22]. Study characteristics are summarized in Table 1. Nine studies included 1326 patients: 602 were treated with dust and 724 were treated with fragmentation. Figure 1 shows the PRISMA flow diagram. Table 2 summarizes the stone and laser basic information. Table 3 summarizes the outcome measures for the two surgical methods, dust removal, and fragmentation. Table 4 demonstrates that the relevant characteristics and variables of this study are comparable. There were no statistical differences in age (*P* = 0.32), male sex (*P* = 0.32), female sex (*P* = 0.12), stone size (mm) (*P* = 0.33), left side (n) (*P* = 0.35), and right side (n) (*P* = 0.25) between groups. The definition of postoperative stone-free rate was not the same in both articles. Four studies defined SFR as stone size < 3 mm or ≤ 3 mm at reexamination after surgery, 2 studies defined SFR as < 2 mm or ≤ 2 mm, 1 study defined SFR as ≤ 1 mm and 2 studies defined SFR as the absence of residual debris with no specific value given. The follow-up time and the time to evaluate the stone-free rate were also different in the nine articles. El-Nahas [18] assessed stone clearance (SFR) with unenhanced computed tomography 2 months after surgery. Yildirim [14]assessed stone-free status using USG, KUB, and NCCT 3 months after surgery. Chen was reexamined 1 month after surgery to assess stone-free status [15]. The definition of operative time varied among the included articles, and operative time refers to the time from cystoscopy placement to urinary catheter fixation. The remaining articles did not mention a detailed definition of operative time. Information on all continuous variables from eight articles was presented as means with standard deviations. Chaloff 2010 ’s continuous variables are presented as medians and quartiles. Finally, shutoff’s data were converted to mean and standard deviation using the data conversion table provided by [9].

**Table 1:** Baselines characteristics of included studies

Reference	Country	Type	Age mean(SD)		Gender (Female, %)		Patients(n)		Left/Right		Stone size (mm)		SFR	Follow-up time
			**Group D**	**Group F**	**Group D**	**Group F**	**Group D**	**Group F**	**Group D**	**Group F**	**Group D**	**Group F**		
Chen 2022	China	cohort study	53.23(12.68)	53.95(13.57)	101(67)	100(66)	150	150	81/69	84/66	51.33(58.69) ^a^	48.9(59.24^a)^	≤ 3 mm	1 month
El-Nahas 2019	Kuwait	cohort study	49.2 (13.2)	50.5 (13.4)	23(45)	25(44)	51	56	34/17	30/26	14.5 (5.5)	14.4 (6.9)	NOR	2 month
Golomb 2022	Israel	cohort study	53.5 (14.8)	54 (13.3)	16(32)	21(42)	50	50	30/17	26/24	17.0 (8.8)	12.7 (5.4)	≤ 3 mm	Long term
Humphreys 2018	America	cohort study	54.7 (13.0)	53.9 (16.8)	27(39)	43(52)	68	82	39/29	37/39	11.3 (4.3)	8.8 (3.5)	NOR	6 week
Lee 2016	China	cohort study	58.6(11.0)	56.1(12.3)	30(39)	57(33)	76	172	NA	NA	11.1(5.2)	11.1(4.8)	< 3 mm	1 month
Liao 2023	China	RCT	46.6(12.7)	47.2(13.2)	39(36)	57(46)	106	112	66/40	62/50	13.5 ± 3.8	14.3 ± 3.7	≤ 2 mm	3 month
Mulåescu 2014	Romania	cohort study	NA	NA	NA	NA	40	40	NA	NA	NA	NA	≤ 1 mm	24 h
Schatloff 2010	Israel	RCT	53.88(15.43)	46.41(12.499)	5(16)	7(30)	30	30	NA	NA	11.43(5.39)	10.90(6.37)	< 2 mm	2 month
Yildirim 2022	Turkey	cohort study	50.29(15.98)	44.59(12.28)	8(25)	11(34)	31	32	12/19	15/17	11.03(3.82)	12.03(4.69)	< 3 mm	3 month

*a* a is defined as the surface area of the stone and other values are defined as the maximum diameter or length of the stone, *NOR* No residual fragments, Mean (SD), *Group D* The dusting group, *Group F* The fragmentation group

**Table 2:** Stones and laser information from included articles

**Reference**	**Number of stones**				**Location**	**Type of laser**	**Laser setting**	
	**Group D**		**Group F**				**Group D**	**Group F**
	**S**	**M**	**S**	**M**				
Chen 2022	137	13	134	16	Ureter	Holmium	0.4 J 40 Hz	0.8 J 8 Hz
El-Nahas 2019	26	25	30	26	Kidney	Holmium	0.3–0.5 J 15–20 Hz	1–1.2 J 6–10 Hz
Golomb 2022	NA				Kidney	Holmium	0.2–0.5 J 20–40 Hz	0.8 J 8–15 Hz
Humphreys 2018	NA				Kidney	Holmium	NA	
Lee 2016	NA				Kidney	Holmium	NA	
Liao 2023	52	54	59	53	Kidney	Holmium	0.2- 0.4 J 30–60 Hz	0.8–1.2 J 8–10 Hz
Mulåescu 2014	NA				Kidney	Holmium	NA	
Schatloff 2010	NA				Ureter	Holmium	NA	0.8-1 J 8–10 HZ
Yildirim 2022	16	15	15	17	Kidney	Holmium	0.5 J-20 Hz	1.2 J 8 Hz

*S* Single, *M* Multiple, *Group D* The dusting group, *Group F* The fragmentation group

**Table 3:** Perioperative outcome

Reference	operation time(min)		Stone free(n)		Hospital stay(day)		Fever		Gross hematuria		UTI		OAC		retreatment rate	
	Group D	Group F	Group D	Group F	Group D	Group F	Group D	Group F	Group D	Group F	Group D	Group F	Group D	Group F	Group D	Group F
Chen 2022	37.73(17.92)	37.6(19.14)	113	123	NA		NA		NA		NA		6	3	29	15
El-Nahas 2019	75.8 (29.6)	91.2 (30.2)	30	44	2.5 (1.3)	2.5 (0.9)	3	3	0	1	NA		4	5	12	9
Golomb 2022	47.2 (25.6)	56.0 (19.1)	31	39	1.1 (0.4)	1.8 (0.5)	4	1	NA		4	2	11	4	14	3
Humphreys 2018	35.9(17.8)	67.4(53.3)	40	61	3.0(3.1)	5.9(6.6)	NA		NA		NA		9	16	1	1
Lee 2016	82.1(2.1)	82.5(2.5)	66	153	NA		2	11	2	3	2	11	8	19	NA	
Liao 2023	43.1(11.7)	60.5(13.4)	83	99	1.2(0.5)	1.3(0.6)	4	3	NA		NA		4	4	10	8
Mulåescu 2014	NA		NA		NA		NA		NA		NA		3	3	0	1
Schatloff 2010	NA		26	30	NA		1	1	NA		NA		2	1	3	0
Yildirim 2022	78.43(30.08)	93.23(27.20)	27	21	2.16(0.73)	2.38 ( 1.21)	3	4	NA		NA		NA		NA	

*UTI* Urinary tract infection, *OAC* Over all complication, *D* The dusting group, *F* The basket group

**Table 4:** The demographics of the studies

Variable	Number of studies with available data	WMD/OR	95% CI	*p* value
Age (years)	8	0.89	(-0.85,2.62)	0.32
Gender(male) (*n*)	8	1.13	(0.89,1.43)	0.32
Stone size (mm)	7	0.68	(-0.68,2.03)	0.33
Laterality(Left)(n)	5	1.14	(0.86,1.52)	0.35

*WMD* Weighted mean diference, *OR* Odds ratio, *Cl* Confdence interval

### Assessment of quality

**Fig. 2:**
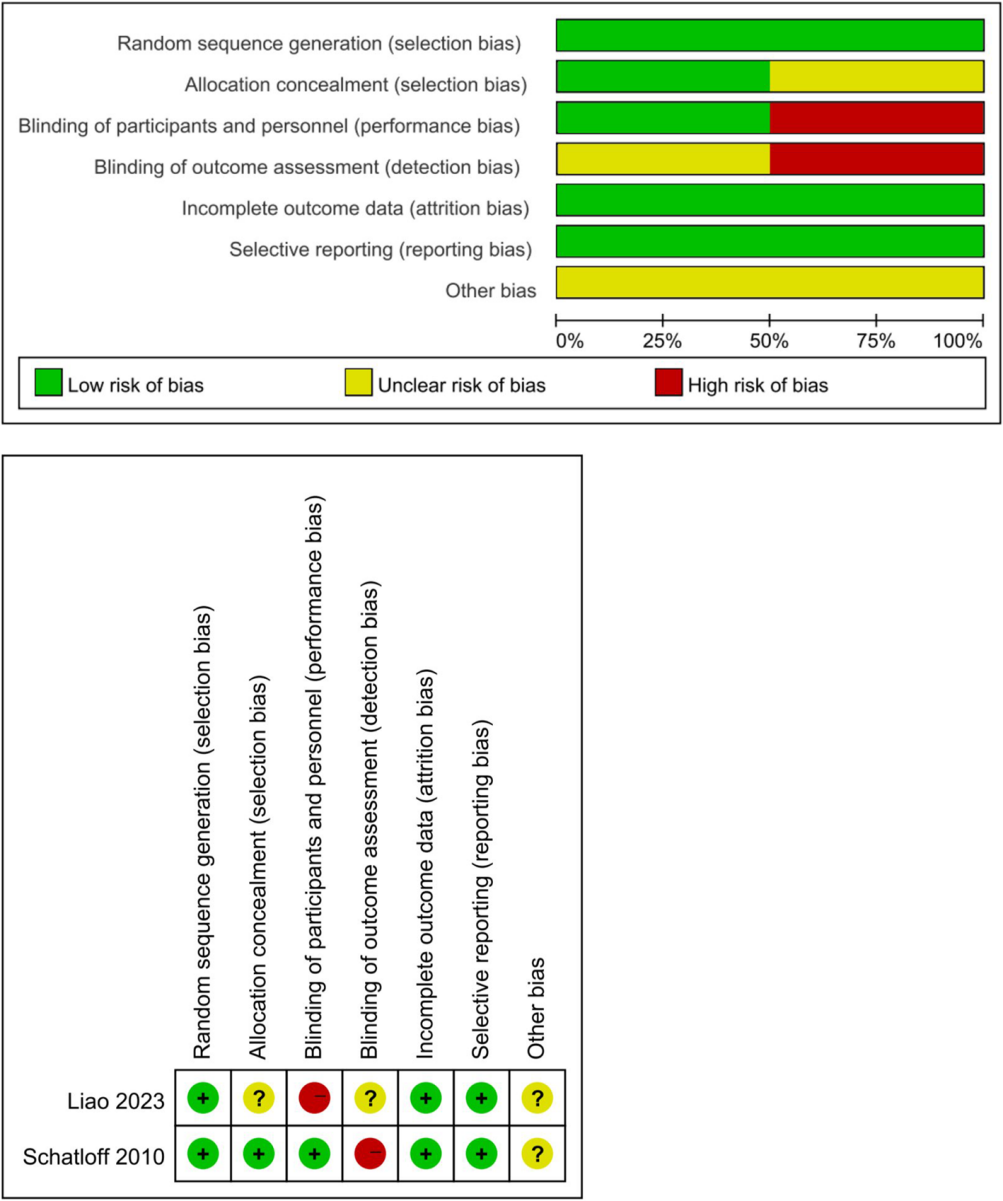
Quality Assessment of Randomized Controlled Studies

Literature NOS ≥ 6 stars in this study was defined as high-quality literature. Two RCT studies had a high risk of selection bias and performance bias due to non-use of allocation scheme concealment and subject blinding [21, 22], and seven cohort studies had literature NOS ≥ 6 stars. Figure 2 shows details of quality assessment for randomized controlled studies. Table 5 shows the details of the quality assessment of the cohort studies.

**Table 5:** Study quality of case–control studies based on the NWewcastle-Ottawa scale

NOS			Selection				Comparability	Outcome			Overall score
**ID**	**Year**	**study_design**	**1**	**2**	**3**	**4**	**5**	**6**	**7**	**8**	
Chen	2022	cohort study	★	★	★	★	★	★	★	★	8
El-Nahas	2019	cohort study		★	★	★	★	★	★	★	7
Golomb	2022	cohort study		★	★	★	★	★	★	★	7
Humphreys	2018	cohort study		★	★	★	★		★	★	6
Lee	2016	cohort study	★	★	★	★	★		★	★	7
Liao	2023	cohort study		★	★	★	★	★		★	6
Mulåescu	2014	cohort study		★	★	★	★	★		★	6
Schatloff	2010	cohort study	★	★	★	★	★	★	★		7
Yildirim	2022	cohort study		★	★	★	★	★	★	★	7

1:Representativeness of the exposed cohor;2: Selection of the nonexposed cohort 3: Assessment of exposure;4:Demonstration that outcome of interest was not present at start of study 5:Comparability of cohorts on the basis of the design or analysis;6: Ascertainment of outcome;7:Long enough follow-up for outcomes to occur;8: Adequacy of follow-up of cohorts

### Surgical outcomes

#### Meta-analysis of stone-free rate

**Fig. 3:**
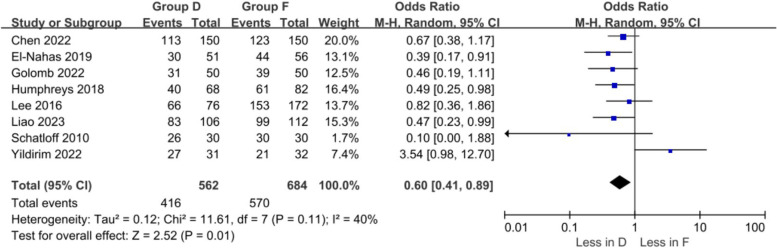
Forest plots of stone-free rate

A meta-analysis of six studies (562 in the dusting group and 684 in the fragmentation group) showed a higher stone-free rate in the fragmentation group (OR 0.6 95% CI 0.41 –0.89, *p* = 0.01). Study heterogeneity was moderate (I^2^ 40%) (Fig. 3). However, in this review, three studies by Humphreys et al. did not specify the laser parameter settings. Laser settings with lower pulse energy (0.2–0.6 J) and higher pulse frequency (50–80 Hz) reduce stone retropulsion and produce small dust-like particles small enough to expel spontaneously or actively. Dust removal relies primarily on gravity-assisted or active suction of dust. Energy, pulse, and frequency were adjusted to achieve optimal power output, and specific parameter settings were empirically adjusted by the surgeon during the procedure [15].

#### Meta-analysis of operation time and re-treatment rate

**Fig. 4:**
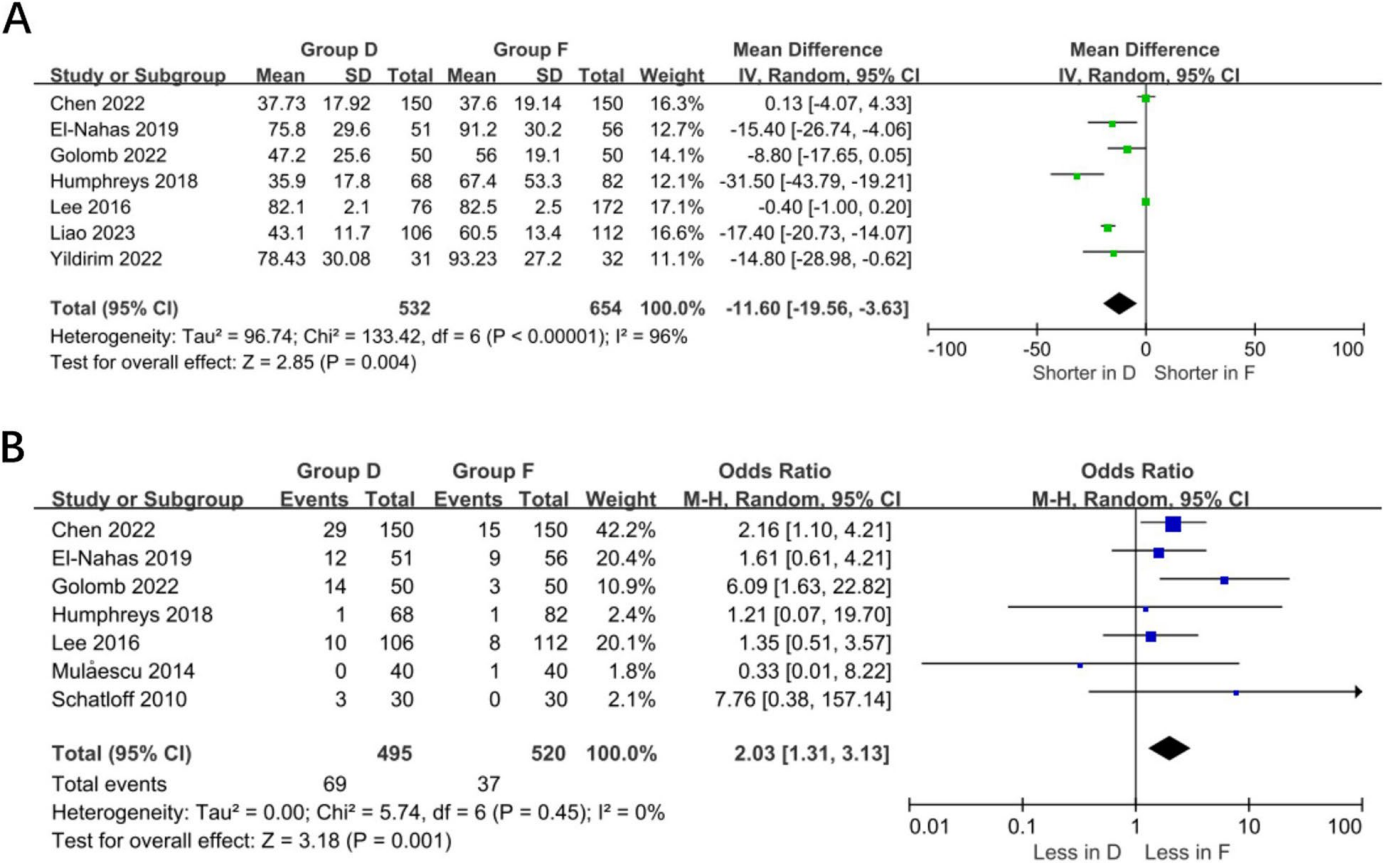
A: Forest plot of operation time. B: Forest plot of retreatment rates

A meta-analysis of 7 studies (532 in the dust group and 654 in the fragmentation group) showed that operative time was shorter in the dust group than in the fragmentation group (WMD – 11.6 min, 95% CI – 19.56 – – 3.63, *p* = 0.004). Study heterogeneity was significant (I^2^ 96%). A meta-analysis of 7 studies (495 dust and 520 fragmentation) showed a higher retreatment rate in the dust group (OR 2.03 95% CI 1.31 –3.13, *p* = 0.001), with insignificant heterogeneity of studies (I^2^ 0%) (Fig. 4).

#### Meta-analysis of fever, length of hospital stays, and overall complications

**Fig. 5:**
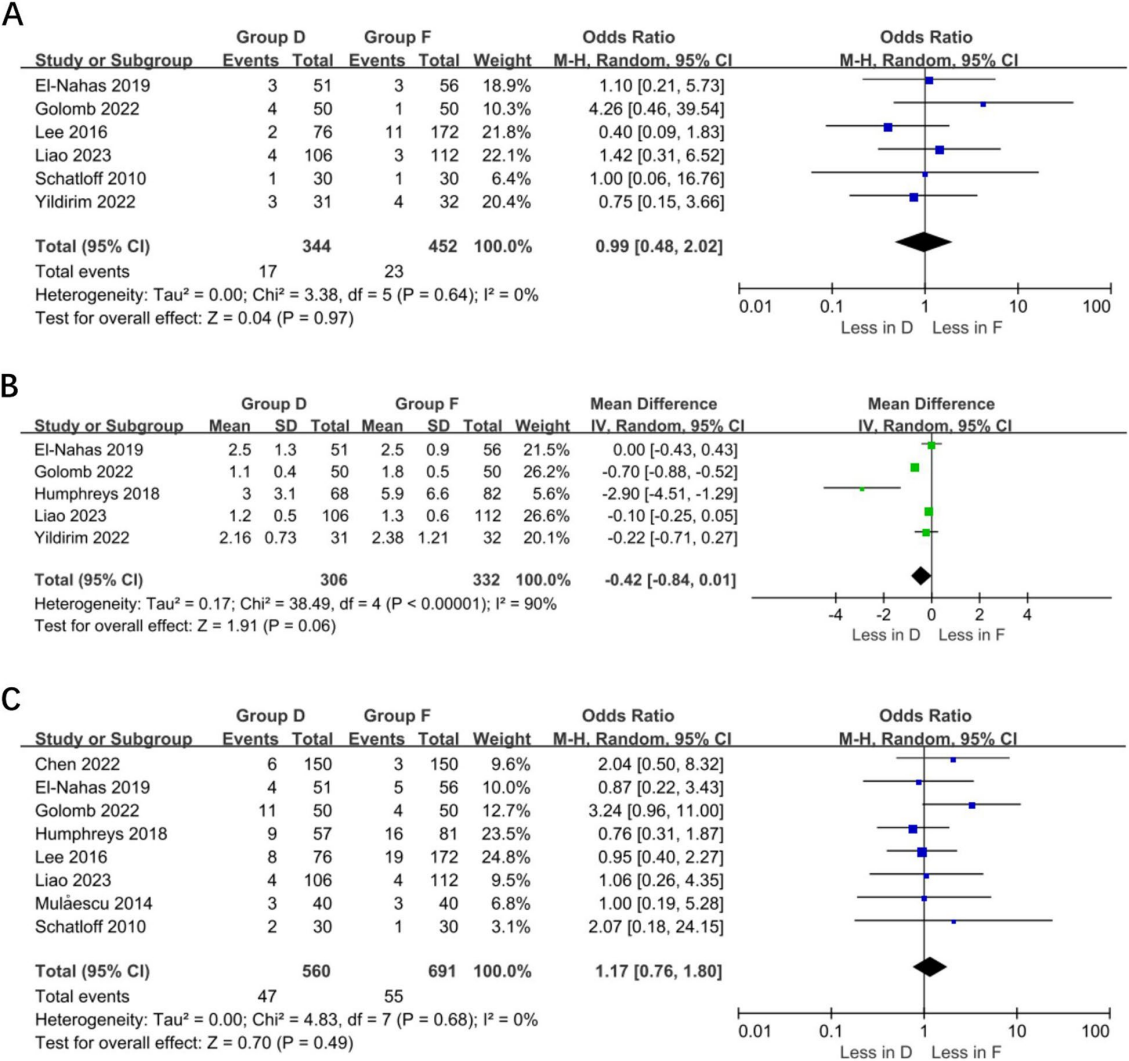
A: Forest plot of fever. B: Forest plot of Hospital stay. C: Forest plot of Overall complication

A meta-analysis of six studies (344 dust and 452 fragmentation) showed no significant difference in the incidence of postoperative fever between the two surgical methods (OR 0.99% CI 0.48–2.02, *p* = 0.97). Study heterogeneity was not significant (I^2^ 0%). A meta-analysis of 5 studies (306 dust and 332 fragmentation) showed no significant difference in hospitalization rates between the 2 surgical methods (WMD—0.42 min, 95% CI—0.84–0.01, *p* = 0.006); study heterogeneity was significant (I2 90%). A meta-analysis of eight studies (560 dust and 691 fragmentation) showed no significant difference in overall postoperative complications between the two surgical methods (OR 1.17% CI 0.76 – 1.80, *p* = 0.49). No study heterogeneity was evident (I^2^ 0%) (Fig. 5).

### Sensibility analysis

**Fig. 6:**
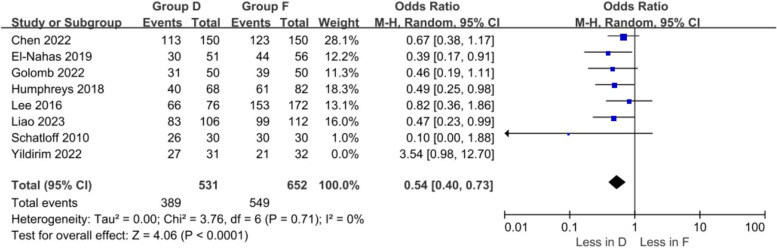
Forest plots of stone-free rate (Excluding Yildirim 2022 Literature)

In this meta-analysis, because some of the results (stone-free rate, operative time, postoperative fever rate) were highly heterogeneous, we performed sensitivity analyses on target parameters to obtain stable and convincing conclusions. The sample size was recalculated using a leave-one-out approach, which showed that the heterogeneity of the stone-free rate significantly decreased after removing one study (Yildirim 2022) (OR 0.54 17% CI 0.40 – 0.73, *p* < 0.0001), and its outcome still indicated that the stone-free rate was higher in the fragmentation group, and the remaining results were stable (Fig. 6).

## Discussion

Advancements in surgical instruments have significantly improved the safety and effectiveness of retrograde intrarenal surgery (RIRS). These advancements include the use of smaller ureteral access sheaths (UAS), intraoperative fluid control devices, and high-power lasers [23]. Ureteroscopic lithotripsy, in particular, has become a preferred treatment method for upper urinary tract lesions due to its minimal invasiveness, fast recovery time, and high stone clearance rate. The use of holmium lasers in ureteroscopy has revolutionized the field by enabling stone fragmentation, stricture incision, and wider access to the ureterorenal system, leading to improved treatment outcomes. However, the high cost of the holmium laser device and prolonged lithotripsy procedures remain a challenge [24]. To optimize outcomes, strategies that consider stone volume and hardness are critical. All studies included in our review used the holmium laser, which can fragment stones either into pieces that can be removed directly with a basket or into fine dust that can be spontaneously discharged ("dusting"). In comparison to the other lasers, the holmium laser (Ho: YAG) is the most commonly used source of URSL because it can fragmentation of any composition calculi and has excellent safety [25]. Holmium laser power is highly absorbed in water with low penetration depth, which means that lithotripsy with holmium laser causes little damage to surrounding tissues [26]. Based on the use of different combinations of energy and frequency, stones in any section can be broken or dusted. The fragmentation setting for the holmium laser was typically between 0.8—1.2 J and 6–10 Hz, while the dust removal setting was between 0.3–0.5 J and 15–60 Hz, The fragmentation group generally used lower frequency and higher energy, while the dust group used higher frequency and lower energy. It has been shown that different settings may have different effects on stones. The higher the pulse energy used, the more retropulsion of the stone occurs, whereas frequency changes have the least effect on retropulsion when energy and fiber diameter remain constant [27]. In this study, further analysis was not performed because there were little data on reported stone retropulsion. Although some studies have shown no significant correlation between the choice of laser mode and stone clearance rate [15], this needs to be further confirmed by more large-sample studies.

### Stone-free rate

Achieving the highest stone clearance rate (SFR) with minimally invasive treatment in a single procedure has become a primary objective of all stone extraction procedures. From the patient’s perspective, the absence of any residual stone after a single operation is more favorable than waiting weeks to expel the stone fragments, even if the remaining stone is small. Factors that affect the final surgical outcome include the available infrastructure, surgeon’s experience, and stone characteristics (size, location, stiffness, and number) as well as patient anatomy [28].In this study, the group that used a basket had a higher stone clearance rate compared to the group that used dusting (OR 0.6, 95% CI 0.41 – 0.89, *p* = 0.01). This result was expected since dusting can result in tiny stone fragments accumulating in the ureteral lumen or renal pelvis, creating dust hills that obscure the line of sight and reduce workspace, particularly in the ureter’s narrow lumen. This can lead to impaired maneuverability and underestimation of remaining fragment sizes. In addition, compared with the fragmentation group, the dust group fragmented stones into fragments ≤ 3 mm or even smaller in diameter. However, this did not mean that the stones completely disappeared, and they may form larger stones again over time, which resulted in decreased long-term stone clearance rate. Moreover, the passage of postoperative fragments could lead to discomfort such as renal colic in patients. some studies have indicated that the passage of stone fragments following surgery is related to more emergency department visits and renal colic [29]. The fragmentation group achieved better visualization during the procedure and was able to remove fragmented stones more efficiently with baskets or forceps due to reduced dust interference. Effective stone clearance is associated with higher postoperative stone clearance, which reduces the risk of stone recurrence after discharge. Brain’s review also confirms this conclusion [30]. Nine studies have assessed that the definition and timing of stone clearance are not the same. In Chen’s study, the definition of absence of stones is no residual stones > 3 mm in the affected ureter within 1 month after surgery. Whereas in Humphreys’ study, the time to review was 4–6 weeks, stone free was defined as no residual fragment of any size on KUB or RUS. In Yildirim’s study, an assessment of stone status was performed 3 months after treatment, and USG, KUB, and NCCT were used to determine stone-free status, which was defined as no residual stones ≥ 3 mm. Assessment of stone clearance status is influenced by many factors, whether the timing of assessment, tools, or definition of stone clearance impacts the comparison of the two surgical modalities. However, the results of eight articles except Yildirim showed that the stone-free rate was higher in the fragmentation group than in the dust group, which was also confirmed by our pooled analysis, but this required more trials, and the definition of stone-free rate was more standardized trials for demonstration.

### Operation time

In this study, operative time was shorter in the dusting group compared to the F group (WMD – 11.6 min, 95% CI – 19.56 – –3.63, *p* = 0.004). Study heterogeneity was significant (I^2^ 96%). The main advantage of the dust removal technique is the ability to complete the procedure with a single pass of the FURS over the wire. The frequency of UAS use in the fragmentation group was higher than that in the dust removal group to facilitate better use of aids such as baskets, and studies have shown that stone fragments removed after lithotripsy require the use of aids such as nitinol baskets to remove residual fragments resulting in prolonged operation time and increased operation costs [18]. However, some relevant meta-analyses pointed out that there was no significant difference in operation time between the dust removal group and the fragmentation group, considering that the time to dust the stones and the time to remove the stones were almost consistent, and the two articles concluded that the different results may be that the quality of the former articles was not high, including many conference abstracts, and this article included the latest cohort study and RCT study [21]. Exactly which surgical method is associated with shorter operative times requires more randomized trials for more standardized comparisons.

## Complication

In this study, the dusting group had a shorter operative time compared to group F (WMD – 11.6 min, 95% CI – 19.56 – –3.63, *p* = 0.004), although study heterogeneity was significant (I^2^ 96%). The main advantage of the dust removal technique is its ability to complete the procedure with a single pass of the FURS over the wire. The fragmentation group had a higher frequency of UAS use than the dust removal group to facilitate better use of aids such as baskets. Studies have shown that stone fragments removed after lithotripsy often require the use of aids such as nitinol baskets to remove residual fragments, resulting in prolonged operation time and increased operation costs [30]. However, some relevant meta-analyses have pointed out that there is no significant difference in operation time between the dust removal group and the fragmentation group, considering that the time to dust the stones and the time to remove the stones were almost consistent. The two articles concluded that the different results may be due to the lower quality of the former articles, including many conference abstracts. This article included the latest cohort study and RCT study, but more randomized trials are required for standardized comparisons. The retreatment rate was higher in group D than in group F (OR 2.03, 95% CI 1.31 – 3.13, *p* = 0.001), and there was a correlation between retreatment rate and stone clearance rate. The lower the stone clearance rate, the corresponding increase in retreatment rate. UAS placement was more common in Group F than in Group D, as shown in many studies. Placement of an introducer sheath in the ureter before RIRS protects the ureter from abrasion from multiple device passes and reduces the chance of postoperative complications. Additionally, UAS placed in the ureter can improve the quality of visualization with continuous irrigation at lower pressure level [31]. The placement of UAS can also reduce intrarenal pressure, which is one of the main reasons bacteria in the collecting system enter the bloodstream through the renal pelvis venous return. This means that the use of UAS in the fragmentation group resulted in a relative reduction in the incidence of postoperative fever, sepsis, and other complications [32]. El-Nahas reported two intraoperative complications of ureteral perforation (grade 3 injury) in Group F, both observed during the removal of the UAS. Traxer and Thomas’ report showed a 46.5% risk of ureteral wall injury with UAS placement, which may be related to preoperative ureteral stent placement, intraoperative surgeon experience, etc [33]. A recent review showed an overall risk of sepsis of 5% following RIRS, identifying risk factors such as whether a stent was implanted preoperatively, the results of urine culture, and the length of operation. Shortening the operation time is beneficial to reduce the occurrence of postoperative sepsis, especially for patients with positive urine cultures after surger [34].

We analyzed postoperative complications by comparing the dusting and fragmenting methods and found no statistical difference between the two lithotripsy techniques in terms of postoperative fever and overall complications. However, no further meta-analysis was performed due to the paucity of studies reporting on other complications or unspecified inclusion in the literature.

### Hospital stay

A meta-analysis of 5 studies (306 dusts and 332 fragmentation) showed no significant difference in hospitalization rates between the two surgical methods (WMD—0.42 min, 95% CI -0.84–0.01, *p* = 0.006). All included articles had shorter hospital stays. It has been shown that RIRS can be used as a day procedure, as long as the procedure is successful and without complications, regardless of the technique used [17].

## Limitations

This study has several limitations that need to be considered. Firstly, due to the lack of data on stone location and characteristics in the included studies, it is difficult to determine which technique is best suited for specific stone types or locations. Secondly, while all studies used Holmium lasers, individual surgeons may have adjusted laser settings, potentially introducing variability in the results across studies. Additionally, the definition of stone-free rate and follow-up time, as well as the imaging tools used, varied among the included studies. Furthermore, no analysis of the cost of the two surgical methods was conducted, which could be a crucial factor in decision-making for physicians and patients. This study did not conduct a subgroup analysis based on stone composition, which is important because different stone compositions can lead to varying efficiencies of lithotripsy. This can somewhat affect the results of the comparison between the two surgical methods. For example, common calcium oxalate stones have a harder texture compared to calcium phosphate stones. This leads to increased lithotripsy time for calcium oxalate stones but also affects the stone clearance rate. Unfortunately, because this article included fewer studies and was not examined for publication bias, its findings may not be fully representative of the entire population of patients with calculi. Finally, the study included fewer randomized controlled trials for direct comparison of the two surgical modalities, and further larger-scale randomized trials are necessary to provide more robust evidence.

## Conclusions

For stones ≤ 20 mm, the dusting method was more potentially advantageous in terms of operative time, but inferior to the fragmentation method in terms of stone-free rate and retreatment rate. However, each technique has relative advantages, and which technique is better depends on the actual situation of the patient and the stone. Currently, more high-quality trials are needed to further evaluate the treatment effect between the two, and in the absence of high-quality randomized controlled trials, urologists must explain the advantages and disadvantages of each laser lithotripsy technique to patients.

## Data Availability

The original contributions presented in the study are included in the article material, further inquiries can be directed to the corresponding author/s.
